# In this issue: Volume 113, Issue 8, August 2022

**DOI:** 10.1111/cas.14958

**Published:** 2022-08-07

## Integration of human inspection and artificial intelligence‐based morphological typing of patient‐derived organoids reveals interpatient heterogeneity of colorectal cancer

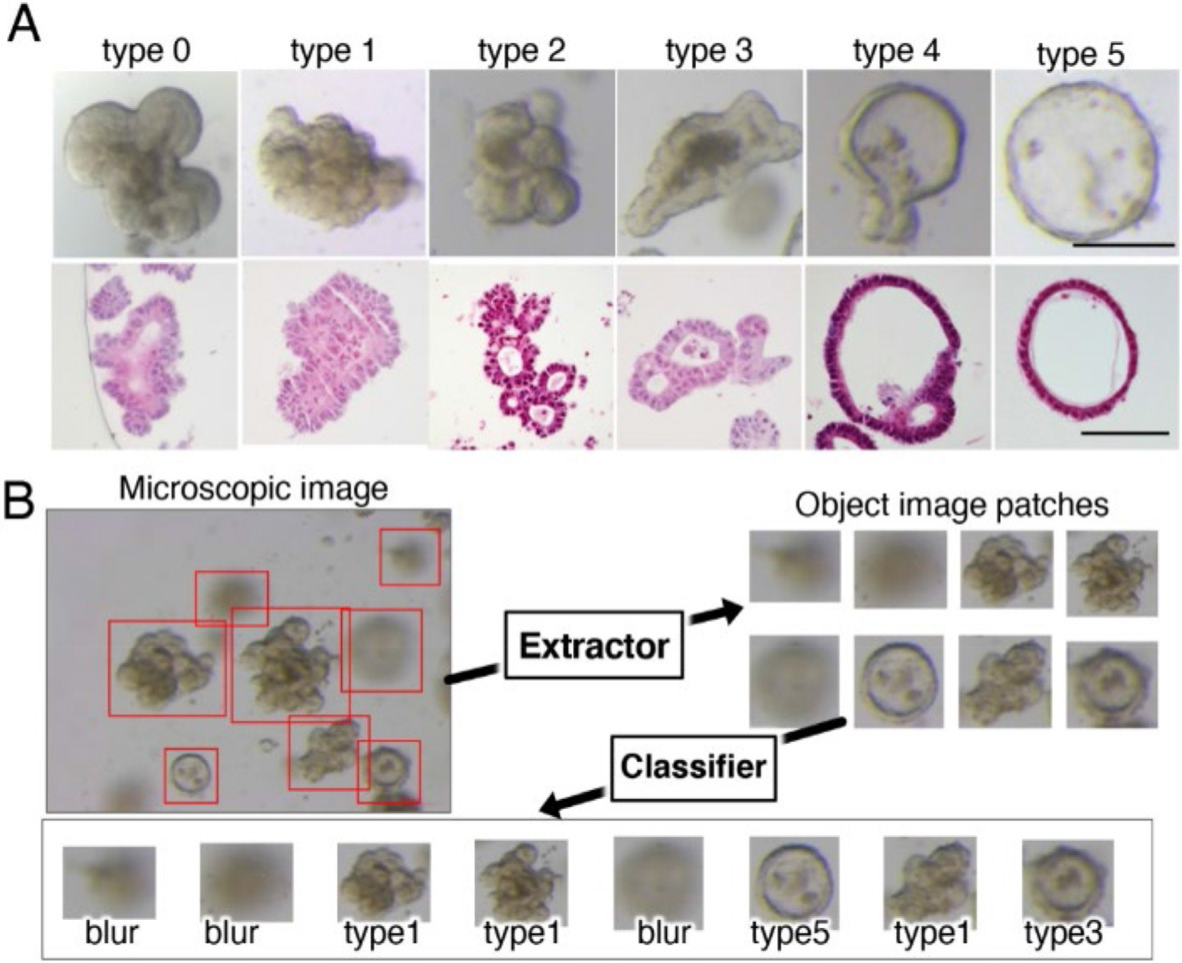

Colorectal cancer (CRC) is one of the leading causes of cancer‐related deaths worldwide. Since it is a heterogenous disease, patients with CRC have differences in tumor morphology, and may not respond similarly to the same treatment regimen. Hence, additional information regarding unknown tumor characteristics is required, for the development of more effective and individualized treatments.

To observe tumor characteristics, researchers often study patient‐derived organoids (PDOs)—in‐vitro culture systems derived from adult stem‐cells in cancerous tissues—which retain the properties of the original tumors.

Recent studies indicate that the morphological features of PDOs derived from metastatic CRC tumors vary considerably. Due to the lack of criteria to classify these features, the biological significance of these PDOs and differences between PDO types have not been established yet.

In this study, Okamoto et al. developed an artificial intelligence (AI)‐based classifier that could classify CRC PDOs based on their morphological differences. To do so, they provided the AI system with 8417 microscopic pictures of 72 PDOs from 26 patients with CRC. Using the authors’ inputs, the AI system classified the PDOs into 6 types (Types 0, 1, 2, 3, 4 and 5), based on similarities in their structures.

They found that most original CRC tumors were tubular adenocarcinomas, classified as “tub1” or “tub2”, based on their degree of differentiation. In addition, genetic analyses revealed that all types of PDOs had genes for cell‐adhering proteins such as integrin, albeit with varying levels of expression, indicating the potential role of these genes in the tissue architecture and development of PDOs.

Interestingly, the morphological characteristics of PDOs did not correlate to the clinicopathological features of the original tumors, which could be explained by the fact that while most tumors grow in different microenvironments, PDOs are grown in identical cultures.

Furthermore, the authors found that PDO types exhibiting higher "stemness", i.e., a higher self‐renewal capability, had higher levels of ribosome biosynthesis genes than those with lower stemness. The former types were also more sensitive to treatment with “CX‐5461”—an inhibitor of ribosomal DNA transcription—which displays anti‐tumor effects against several types of cancer cells. These findings imply that PDO morphology is a significant predictor of patient response to chemotherapy.

To conclude, the AI classifier successfully categorized PDOs and identified the differences in tumor characteristics between patients. By evaluating these differences, clinicians can accurately determine the type of treatments that will be effective for patients with different CRC tumor morphologies.

https://onlinelibrary.wiley.com/doi/full/10.1111/cas.15396

## A tumor metastasis‐associated molecule TWIST1 is a favorable target for cancer immunotherapy due to its immunogenicity

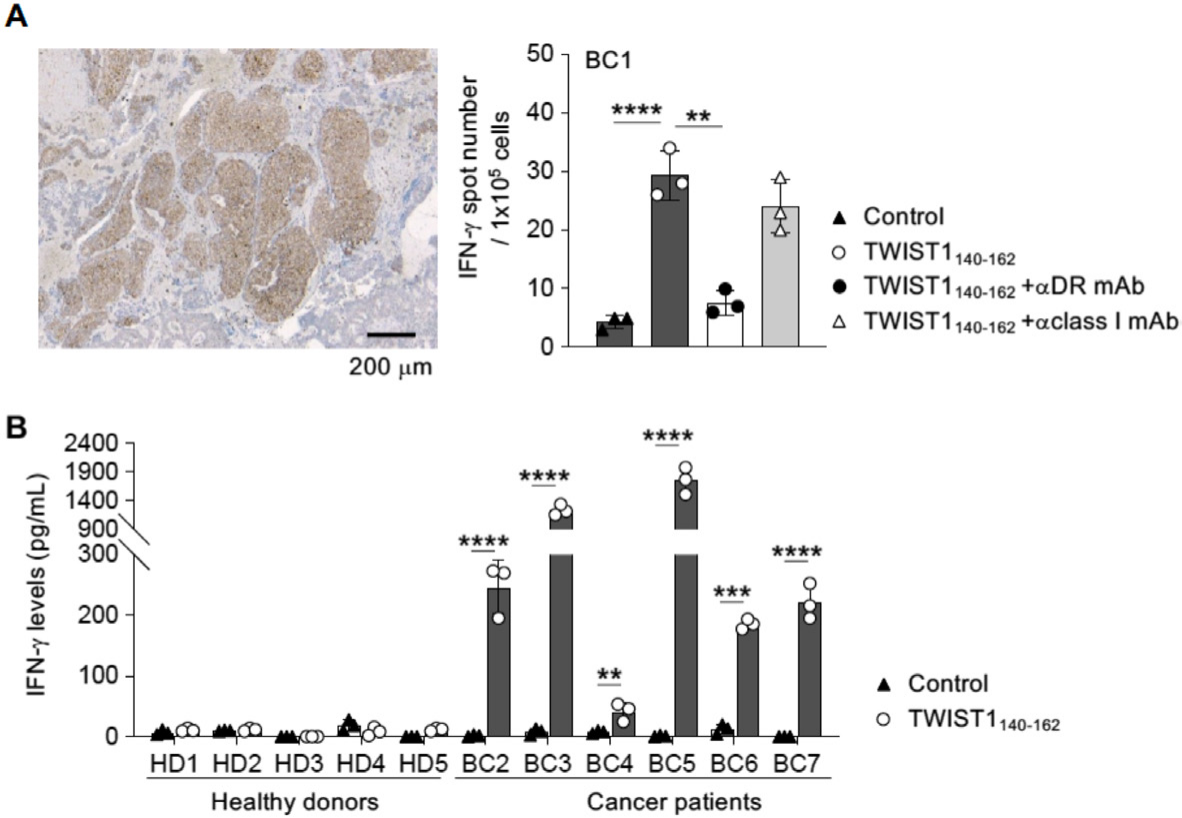

Immunotherapy is a cancer treatment strategy that involves stimulating the immune system to identify and attack cancer cells. This is achieved by targeting specific proteins on cancer cells to make them available for immune recognition and activity using antibodies and immune cells.

Cancer cells often possess significantly mutated genes, causing them to express modified versions of proteins that exist normally in other cells. These modified proteins, called ‘neoantigens,’ are the favorable targets of different immunotherapy strategies although their clinical use is costly.

Neoantigens are highly immunogenic, i.e., easily recognized by the immune system. Most cancer cells only express them in the later phases of cancer progression, when an immune suppressive microenvironment has been established. However, neoantigens vary among individuals, which is a disadvantage while developing neoantigen‐based immunotherapy strategies.

Highly immunogenic antigens among tumor‐associated antigens (TAAs), can also be upregulated in cancers in the later phases. TAAs are also broadly expressed across several types of tumors, making them a more favorable target for immunotherapy.

One such TAA is the protein Twist1, encoded by the TWIST gene, which is highly conserved from invertebrates to humans. It is strongly expressed in a range of cancers in their later stages and plays an important role in metastasis of the tumor. Twist1 is hypothesized to be a favorable target for immunotherapy.

In this study, Yajima et al. evaluated the immunogenicity of the Twist1 protein and its potential as an immunotherapy target. They tested different sections of the Twist1 protein using synthetically manufactured peptides on freshly isolated immune cells such as T‐cells and monocytes, and identified a region of the protein, TWIST1_140–162_, that activated T‐cells into CD4^+^ helper T‐cells (HTLs). These HTLs recognized Twist1 expressing cancer cells and released interferon gamma (INFγ), a key messenger of the anti‐cancer response, highlighting the immunogenicity of Twist1.

The authors then tested if Twist1 expressing tumors could naturally elicit the formation of HTLs in cancer patients. They observed that HTLs isolated from breast cancer patients underwent rapid activation and expansion on exposure to TWIST1_140–162_. The frequency of such cells was found to be significantly higher when compared to healthy individuals, confirming that the high levels of Twist1 expressed in tumors lead to the development of HTLs against it. Additionally, they also found that vaccinating mice with the peptide TWIST1_140–162_ led to the development of Twist1 specific HTLs and that combining this with other immunotherapy treatments like immune checkpoint inhibitors significantly improved anti‐tumor HTL activity.

These findings confirm that Twist1 is an immunogenic protein present on cancer cells, which could be targeted effectively to treat metastatic tumors in patients with cancer.

https://onlinelibrary.wiley.com/doi/full/10.1111/cas.15429

## Oxidized high mobility group B‐1 enhances metastability of colorectal cancer via modification of mesenchymal stem/ stromal cells

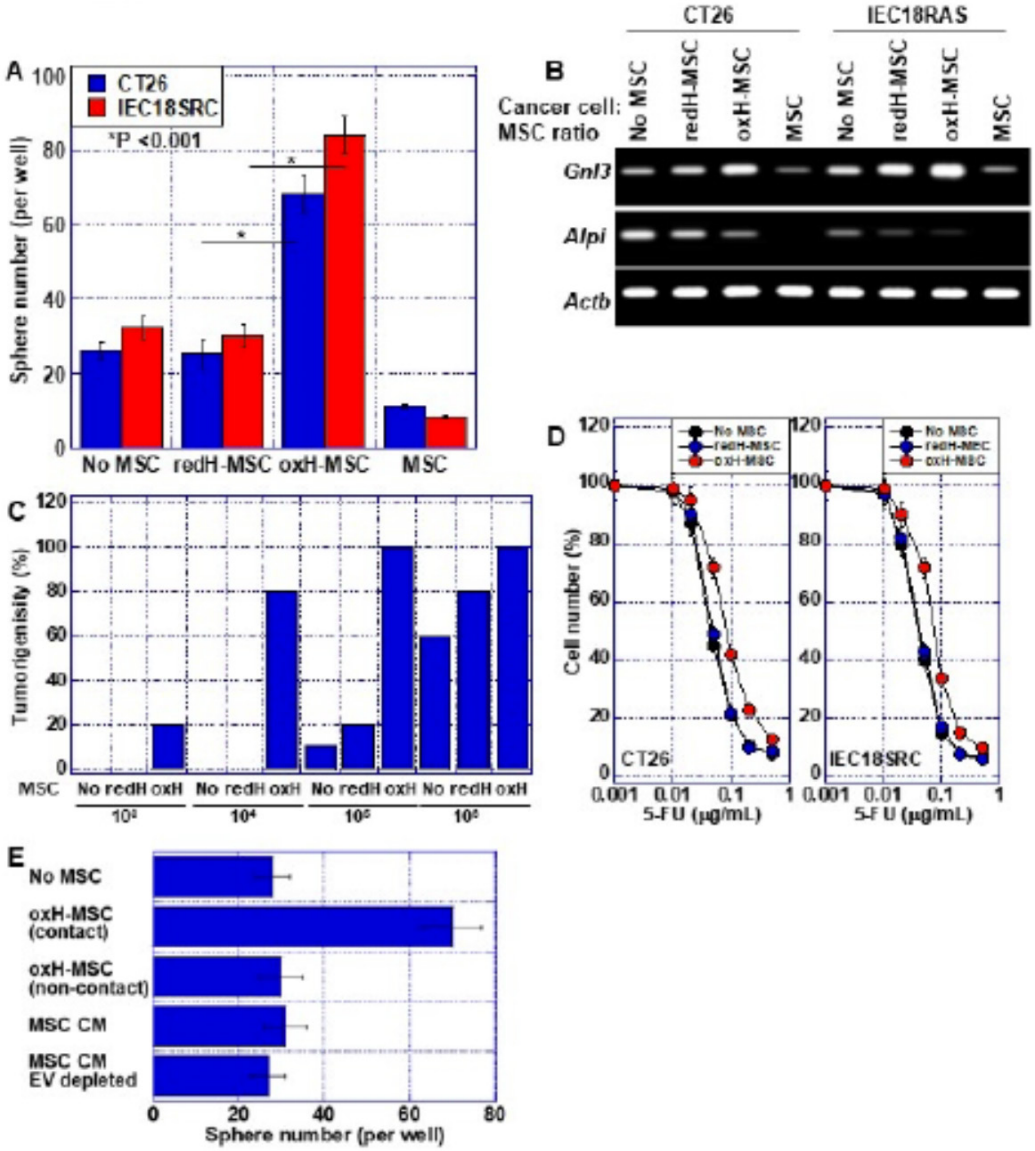

Colorectal cancer (CRC) is one of the leading causes of cancer‐related deaths worldwide. With a growing number of cases reported each year, CRC stage‐4 cases have a poor 5‐year survival rate of only about 19%. Moreover, nearly one‐third of all CRC deaths are directly related to liver metastasis, which is present in around 25% of advanced CRC cases.

The self‐renewing mesenchymal stem/stromal cells (MSCs) have been isolated from many cancer tissues and have a role in the proliferation of cancer. The rapid growth and movement or chemotaxis of the MSCs is controlled by a group of nuclear proteins called high mobility group box‐1 (HMGB1). The HMGB1 proteins are known to have a significant role in governing cellular responses to infection, injury, and inflammation, but their exact role in cancer progression is uncertain.

It has been previously established that the function of HMGB1 is altered by post‐translational modifications, including oxidation. Do differences in the oxidation state of HMGB1 have a significant impact on the function of MSCs? There are suggestions that the reduced and oxidized forms of HMGB1 may have contrasting functions and therefore varied roles in CRC.

To know more, Kishi et al. evaluated the effects of HMGB1 on bone marrow MSCs (BM‐MSCs) by conducting a series of molecular investigations on human as well as mice colon cancer cells. The team established cell cultures and suitable tumor models to explore the modified action of HMGB1 following post‐translational modifications.

The authors found that in its reduced state, HMGB1 inhibited the build‐up of BM‐MSCs in mice and activated their differentiation into other cells like bone cells or cells of blood vessels. Conversely, oxidized HMGB1 promoted the proliferation of BM‐MSCs, leading to their rapid growth but no differentiation.

In humans, the number of MSCs and HMGB1 levels have been correlated with liver metastasis. Supporting this notion, the authors found that oxidized HMGB1 indeed promoted liver metastasis in an induced‐CRC mouse model by increasing the movement of MSCs into the intra‐tumoral region.

These findings confirm that while reduced HMGB1 inhibits MSCs, oxidized HMGB1 boosts the "stemness" of cancer cells. It does this by reprogramming MSCs to promote cancer, which in turn accelerates the proliferation of cancerous cells. Oxidized HMGB1 may thus serve as a potential target for CRC therapy.

https://onlinelibrary.wiley.com/doi/full/10.1111/cas.15400

